# Metacognitive Feelings as a Source of Information for the Creative Process: A Conceptual Exploration

**DOI:** 10.3390/jintelligence11030049

**Published:** 2023-02-28

**Authors:** Rogelio Puente-Díaz

**Affiliations:** School of Business and Economics, Universidad Anáhuac México, Av. Universidad Anáhuac 46, Huixquilucan C.P. 52786, Mexico; rogelio.puente@anahuac.mx

**Keywords:** metacognition, creativity, metacognitive feelings, creative process, feelings-as-information, inferential processes, divergent thinking problems

## Abstract

Philosophers and psychologists have debated the wisdom of using feelings as a source of information when making decisions. While not trying to solve this debate, a complementary approach is to examine how metacognitive feelings are used when generating, evaluating, and selecting ideas to solve creative problems and whether their use leads to accurate idea evaluation and selection. Hence, this conceptual article aims to explore how metacognitive feelings are used to evaluate and select creative ideas. Interestingly, while metacognitive feelings come from the perceived ease or difficulty of generating solutions to creative problems, these feelings also inform the decision to continue generating ideas or stop. Metacognitive feelings are then an integral part of the creative process of generating, evaluating, and selecting ideas. The present article briefly reviews the history of metacognitive feelings as examined in metamemory, meta-reasoning, and judgment formation in social psychology, before discussing their implications and potential for understanding the creative process. The article ends by positing directions for future research.

## 1. Introduction

The examination of metacognition is an exciting field of inquiry with multiple contributions from meta-memory (e.g., Dunlosky and Metcalfe 2009; Koriat 2012), meta-reasoning (e.g., Ackerman and Thompson 2017), decision-making (e.g., Evans 2020; Stanovich 2011), social psychology (e.g., Schwarz 2018), educational psychology (Moshman 2015; Preiss 2022; Tarricone 2011), and creativity (Beghetto and Madison 2022; Kaufman and Beghetto 2013; Karwowski et al. 2020). As is often the case, when there is widespread excitement, there is also confusion as to what metacognition is, its main components, and its utility as a construct (see Kuhn 2022 for a recent discussion). Recent conceptual efforts have tried to develop a model to examine metacognition in creativity research (Lebuda and Benedek Forthcoming), which should provide a guiding framework for future research on creative metacognition. While the main goal is not to help eliminate this confusion, a general overview is offered of how metacognition has been examined in the different research domains and place special emphasis on one specific component, metacognitive feelings, and their role in the creative process (see Efklides 2006 for one of the first discussions of metacognitive feelings). I define metacognitive feelings as the feelings coming from the act of thinking (Efklides 2006; Vogl et al. 2021). Hence, the purpose of the present article is threefold. First, I present a conceptual development of how metacognitive feelings inform the creative process. Second, I review studies that have empirically examined the influence of metacognitive feelings on different components of the creative process. Third, I speculate about the intelligent use of metacognitive feelings in terms of greater production of creative ideas and accurate idea evaluation and selection. In order to achieve the research goals, I need to first define the type of problems examined in creativity research, and what creativity is, before explaining how metacognitive feelings inform the creative process. I conclude the article by providing suggestions for future research.

## 2. Conceptual Definitions of Creativity and Types of Problems

Creativity scholars suggest adopting a dynamic definition of creativity where the observed originality and effectiveness of the ideas generated are in constant development and rarely truly finalized (Corazza 2016). When individuals generate ideas to solve a problem, the first judgments of originality and effectiveness represent first impressions likely to evolve as a function of time, further idea development, and self- and other feedback, among other aspects. Previous definitions used similar dimensions, originality and effectiveness, without emphasizing the dynamic nature of judgments of originality and effectiveness (e.g., Stein 1953). Engaging in the creative process involves generating, evaluating, and selecting ideas, but it does not have to end in finalized products. The process of creative engagement represents a valuable process and outcome in itself, which can be examined dynamically. Based on this conceptualization, scholars suggest thinking in terms of potential originality and effectiveness. When defining creativity as a given potential to be achieved, the ideas generated represent important outcomes, but not the only outcomes to be observed. Motivational variables such as effort, persistence, and attributions are likely to play an important role, as well.

Under this dynamic conceptualization of creativity, creativity is defined as something with potential originality and effectiveness. The potential originality and effectiveness are first assessed by idea generators and sometimes, after, by an audience. Metacognitive feelings could have implications for self- and other assessments of potential originality and effectiveness, yet my focus is on self-evaluation and selection.^1^

Even though there seems to be a general understanding that creativity mainly focuses on divergent thinking problems or ill-defined problems, the literature on creativity has not extensively addressed the influence of types of problems on metacognition. Specifically, and making an over-generalization, most problems used in meta-memory and meta-reasoning are problems with well-defined answers and with single correct answers. Conversely, divergent thinking problems do not have single, correct solutions. This difference makes the assessment of one´s solutions to a problem more difficult for divergent thinking problems. Hence, self-assessments could be more inconsistent and less accurate for divergent thinking problems. In addition, while problems with a single, correct solution might also have external validation (a professor or software checking the correct answer), they do not need to. Individuals might self-assess the correctness of an answer with high levels of accuracy. Conversely, the solutions to ill-defined problems almost always need the validation of an audience. This additional validation, which is almost always needed, increases the uncertainty of any judgment of potential originality and effectiveness with important metacognitive consequences. 

Combining the discussion of the types of problems found in most creativity research with the definition of creativity emphasizing potential originality and effectiveness sets the stage for pointing out the main similarities and differences between metacognitive processes in meta-memory, meta-reasoning, judgment formation in social psychology, and creativity. I emphasize the role of metacognitive feelings and focus on suggesting implications for creativity research. 

## 3. Meta-Memory, Meta-Reasoning, Judgment Formation, and Creativity Research

The mind is capable of generating outputs in the form of answers to memory questions, judgments about self-qualities, answers to reasoning problems, and solutions to creative problems. In addition, the mind can also monitor the quality of its own outputs. The distinction between generating and monitoring cognitive processes has been conceptualized as object- and meta-level processes (Nelson and Narens 1990). This distinction has been instrumental to advancing our understanding of metacognition in memory, reasoning, social psychology, and also creativity research, even though it is probably not as developed in creativity research. A central concept of the monitoring process is that individuals do not have direct access to their cognitive operations or outputs. Hence, they use cues to inform their judgments. These cues differ in their level of diagnosticity (Ackerman 2019). In memory, reasoning, and creativity problems, individuals want to provide their best possible answers, yet the process of knowing and inferring when individuals have reached this goal differs as a function of the type of problem. I suggest that problems without a single solution, such as the ones found in creativity, have more uncertainty, making cues less reliable and valid, but it is still important to generate, evaluate, and select creative ideas. A brief review of the problems solved in meta-memory and meta-reasoning research will help illustrate this point, especially the work on memory and reasoning problems, given that it has been the most influential for the development of metacognition in creativity research (Ackerman 2019).

To provide an example, in memory research, one of the most used experimental tasks is to ask participants to study a list of items and then make an estimate of the likelihood of recalling the studied items in a subsequent test (Dunlosky and Metcalfe 2009; Metcalfe 2000). Results have shown that this feeling of knowing shows acceptable levels of accuracy, higher than expected by chance, suggesting that participants know that they will know based, partially, on how they feel while studying a set of items (e.g., Metcalfe and Finn 2008). The task of studying a set of items and recalling them at a later time does not only represent a task with correct answers but also a task where participants have abundant experience. It is a relatively simple task compared to generating solutions to ill-defined problems such as the ones found in creativity. It is also a task with clearer criteria for success than ill-defined problems. Individuals need to determine whether they will be able to recall something correctly. Correctness is a criterion with less ambiguity than generating creative solutions. In addition, it is a task where individuals might feel confident and accurate about their answers even without external feedback from others, reducing uncertainty.

Similarly, in meta-reasoning research, one of the most often used experimental tasks is solving syllogisms, in which participants are asked to estimate the validity of a conclusion based on a set of implicit rules of logic. Individuals should pay attention to the rules of logic without considering whether the conclusion is believable or not. Empirical findings suggest that individuals do pay attention to how believable the conclusion is even when the conclusion is invalid (see De Neys and Franssens 2009 for an empirical study and Ball and Thompson 2018 for conceptual development) because believable conclusions feel right. Even though syllogisms are more difficult and cognitively demanding than simple memorization tasks, they still represent problems with single, correct solutions. The possibility of finding the right solution reduces the uncertainty when solving this type of problem. Correctness is a criterion with less ambiguity than generating creative solutions. 

When focusing on problems with multiple solutions and the creative process, a central question is how idea generators monitor the creativity of their own ideas. In other words, how do idea generators know that the ideas generated are original and effective? The answer to this question has important consequences in terms of effort, time allocation, the decision to continue or stop generating ideas, and ultimately, the difficult decision of choosing to pursue a given idea. Individuals have to answer similar questions when trying to recall something (meta-memory), when reasoning about the validity of a conclusion (meta-reasoning), and when self-evaluating how assertive they are by recalling examples of assertiveness (ease of recall) from their personal past. The main differences, as explained earlier, are that creativity problems have more uncertainty and more ambiguous criteria. Consequently, the work on education, memory, reasoning, decision-making, and social psychology has served to develop our understanding of how metacognition functions with the main difference that creative problems do not have single, correct solutions and have more ambiguous evaluative criteria. Creativity scholars have used previous work on metacognition to draw implications for creativity research (see Kaufman and Beghetto 2013), yet more emphasis is needed on how divergent thinking problems pose additional challenges. 

Going back to the basic question that idea generators have to answer about the potential originality and effectiveness of the ideas generated, I point out that individuals rely on different available sources of information to make such inferences (Koriat 2006). A source of information is something that individuals use and that often correlates, positively or negatively, with idea generation, evaluation, and selection. The extent of the correlations and the degree of accuracy are often a function of the diagnosticity of the source of information (Ackerman 2019). Some sources are going to be more reliable and valid than others. Their level of reliability and validity is a matter of degree, without any source being perfectly reliable and valid in most cases and situations. The level of reliability and validity is likely to vary as a function of different aspects, but one of the most important ones with implications for creativity is the type of problem to be solved. Specifically, the validity and reliability of different sources of information should be low when solving divergent thinking problems for the simple reason that they do not have a single, correct solution. Thus far, most research has examined metacognition when solving problems with single, correct solutions. This is an important shortcoming, especially for creativity research, because problems without a single solution pose different, probably more difficult challenges. I posit that metacognitive feelings are a source of information that can inform on the degree of creativity of the ideas generated. 

## 4. How the Different Components of Metacognition Interact

**Figure 1:**
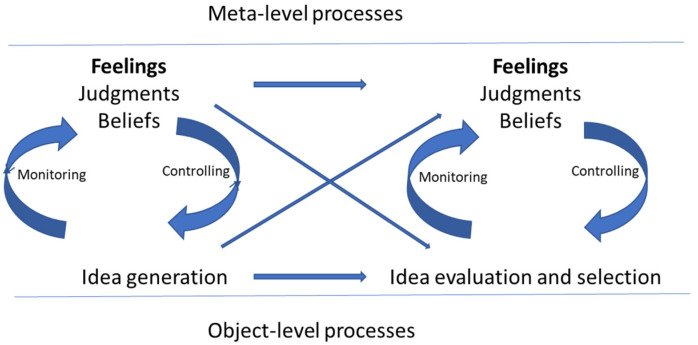
How feelings inform the creative process.

Metacognition scholars suggest three facets of metacognition: knowledge, experiences, and monitoring and control (Flavell 1979). Metacognitive feelings are located within the experiences facet of metacognition (see Efklides 2006 for one of the first propositions of locating feelings as an indicator of the experience facet of metacognition). Similar models have been proposed recently for creativity research (Lebuda and Benedek, forthcoming). I use a model developed in social psychology (Unkelbach and Greifeneder 2013) to specify how feelings influence the creative process. This model suggests that individuals need to assess the potential originality and effectiveness of their ideas as an indicator of creative potential. Metacognitive feelings represent one way of monitoring the potential originality and effectiveness of ideas. The problem is that the level of potential originality and effectiveness of ideas is a distal criterion. It is a criterion that idea generators do not have direct access to, and it takes time to develop the skills needed to make such judgments. It is also a criterion that, in most cases, needs social validation, that is, validation from others. Given the lack of access to this distal criterion, idea generators need to rely on proximal cues as a source of information. One of these cues is metacognitive feelings in the form of ease of idea generation and feelings about the quality of ideas. These feelings serve as imperfect cues, indicating that the ideas might be creative because: “I feel good about them”, “They are coming with ease”, or “I felt at ease while generating these ideas” (see Koriat 2006 for a conceptual discussion of feelings as a source of information). Hence, feelings might influence the process of idea generation, evaluation, and selection even in situations where they might not be the most valid and reliable cue (see Figure 1 for a visual representation of the conceptual development). Other cues, besides feelings, might include beliefs about the creative process and confidence in the ability to generate and evaluate ideas, among others.

Given the interaction between metacognitive knowledge, experiences, and monitoring and control, it is likely that metacognitive feelings influence and are influenced by metacognitive knowledge, control, and monitoring. Hence, I posit that knowledge, experiences, and monitoring and control are part of creative metacognition. Consequently, I expect different indicators of metacognitive knowledge, experiences, and monitoring and control to relate to each other (see Lebuda and Benedek Forthcoming for a similar conceptualization). Empirical studies have focused on testing some of these relationships.

## 5. Knowledge and Experiences: Creative Self-Efficacy and Metacognitive Feelings

Creativity scholars have paid attention to creative self-efficacy as a relevant self-belief for the creative process, given its positive relationship with the originality and effectiveness of the ideas generated (Beghetto and Karwowski 2017). The relationship between creative self-efficacy and metacognitive feelings can take two forms: creative self-efficacy as an antecedent of metacognitive feelings, and metacognitive feelings serving as a source of information about creative self-efficacy. Both directionalities have been tested. 

For example, in one empirical study, researchers manipulated ease of recall, a form of metacognitive feeling, by asking participants to bring to mind a few versus many examples of creative idea generation from their personal past (Puente-Díaz and Cavazos-Arroyo 2020). The core idea is that participants who brought to mind fewer instances of creative idea generation would experience greater levels of ease of recall, which then would be positively correlated with creative self-efficacy and creative potential. Results showed support for this. Participants who recalled fewer instances of creative idea generation reported higher creative self-efficacy because they experienced higher levels of metacognitive feelings and were able to generate more creative ideas on a divergent thinking task than participants who reported many instances. These results were also consistent with the idea that creative self-efficacy is a positive predictor of performance on idea-generation tasks (see Karwowski et al. 2019). The logic behind these findings lies in the availability heuristic (Tversky and Kahneman 1973). Specifically, participants interpreted the ease of bringing to mind a few instances of creative idea generation as an indirect, imperfect indicator of their behavior, which helped define themselves as more confident. Then, this enhanced confidence energized their idea generation. These findings only make sense when adopting a situated cognition approach to human thinking (Schwarz 2015). Situated cognition approaches posit that thinking is for doing. Self-beliefs of efficacy are malleable and informed by cues such as metacognitive feelings coming from ease of recalling instances of creative idea generation. Participants used these feelings to make self-judgments about their ability to generate ideas. These augmented beliefs also served as a source of information when participants were generating ideas, resulting in a greater production of creative ideas.

Regarding the option of creative self-efficacy acting as an antecedent of metacognitive feelings, one study found a positive relationship between creative self-efficacy and metacognitive feelings (Puente-Díaz and Cavazos-Arroyo 2022a). Creative self-efficacy was assessed before introducing participants to the problem to be solved, and metacognitive feelings were assessed after participants finished generating ideas. I interpret this significant correlation as an indication that metacognitive knowledge facilitates the experience of ease while generating ideas. Hence, from both studies, I infer a reciprocal relationship between metacognitive knowledge and experiences, and between creative self-efficacy and metacognitive feelings. 

Given the promising results just explained, future studies could extend the examination of creative beliefs to include creative personality identity and creative mindsets as possible antecedents of metacognitive feelings (see Karwowski et al. 2019 for a discussion of relevant creative beliefs). In addition, metacognitive feelings might also act as a source of information when making judgments about the stability and malleability of creative skills before performing a creative task. Consequently, metacognitive feelings could inform creative mindsets, as well.

## 6. Experiences and Monitoring and Control: Metacognitive Feelings and Idea Evaluation and Selection

The use of feelings as a source of information has been examined in metacognitive research and in research on emotional intelligence (see Agnoli et al. 2019). The proposition is that emotional intelligence skills allow individuals to effectively use feelings to improve performance in the different stages of the creative process. By conceptualizing feelings as a source of information, I suggest that feelings inform the process of evaluating and selecting ideas. Evaluating and selecting ideas could be conceptualized as indicators of the monitoring and control component of metacognition. 

For example, researchers conducted two studies involving the generation of ideas to solve marketing problems and then the evaluation of the ideas generated by participants and judges. In both studies, metacognitive feelings were collected right after the idea-generation task concluded and focused on assessing ease of idea generation (Puente-Díaz et al. 2021a). In study 1, metacognitive feelings had a positive relationship with an indicator of overestimation of the creativity of the ideas generated. Overestimation was conceptualized as the difference between participants’ evaluations of the creativity of their ideas and the evaluations made by independent judges. This overestimation was then negatively related to accurate idea selection. The key interpretation is the following. After generating ideas, participants use any cue available to conduct self-evaluations. Knowing how original and effective self-generated ideas are is a difficult task with uncertainty. Feelings represent an available cue. In this study, it seemed participants used this cue to evaluate their ideas more positively than independent judges did. In study 2, the results showed a positive relationship between metacognitive feelings and the relevance of the strengths and weaknesses identified in the ideas generated. Relevance then had a positive relationship with accurate idea selection. In this study, feelings informed the important process of evaluating ideas by identifying positive and negative attributes, which then helped make the difficult decision of how to choose the most creative idea. In both studies, feelings functioned as a source of information that individuals used to make self-evaluations and identify strengths and weaknesses in the ideas generated.

In another empirical investigation, three studies were conducted examining metacognitive feelings (Puente-Díaz et al. 2021b). In all three studies, metacognitive feelings were positively correlated with the overestimation of the creativity of the ideas generated. In one of these three studies, ease of idea generation had a positive relationship with accurate idea selection. The interpretation again is that participants utilized feelings as a cue to evaluate the creativity of their ideas. Regarding the issue of overestimation of the creativity of ideas, it is worth mentioning the findings from a recent study that showed that participants underestimated the originality of their ideas (Sidi et al. 2020). Sidi et al. (2020) found that participants evaluated their ideas as less original than they really were based on the observed frequencies of responses. While this study made an interesting and relevant contribution, it relied on asking participants about how many participants came up with the same idea without considering the effectiveness of the ideas. In most of the articles reviewed thus far, participants evaluated their ideas in terms of how original and effective they were, often summarized in an overall creativity judgment. A creative idea needs to be original but also effective to be considered creative. In addition, the issue of under- and overestimation has been discussed from the perspective of overconfidence and how evaluating self- and other generated ideas differs (Moore and Healy 2008). Regarding the intelligent use of feelings to choose accurately self-generated ideas, only one study found a positive relationship, suggesting that future studies are needed to establish the robustness of the observed significant relationship. 

In one study examining consequences of metacognitive feelings, the results showed that feelings significantly informed self-evaluations and post-task creative confidence (Puente-Díaz and Cavazos-Arroyo 2022a). What this suggests is that metacognitive feelings were used to inform post-task creative confidence. This is particularly relevant because creative confidence was assessed before and after generating ideas. Pre-task creative confidence predicted metacognitive feelings, and metacognitive feelings helped participants feel more confident about the potential originality and effectiveness of the ideas generated, though important motivational implications, such as persistence and effort in idea refinement, were not explored in the study. 

An interesting twist to the study was the result that the serial order effect found in previous investigations (Beaty and Silvia 2012; Sidi et al. 2020) only held for judges’ evaluations. For self-evaluations, participants evaluated and selected their first ideas as the most creative at higher rates than expected by chance. This last finding could be interpreted as the contribution of ease of idea generation to idea selection, given that it seems reasonable to assume that the first idea felt as if it came with greater ease than the rest of the ideas. For this particular study, using feelings did not help participants accurately select their most creative idea compared to the judges’ selection. A recent study found similar results but had participants make predictions about the creativity of their ideas across time and compared them to the judgments made by others (Lucas and Nordgren 2020). Participants predicted their ideas would decrease in creativity across time when, in fact, they increased. Future studies asking participants to evaluate their ideas should try to replicate these results to assess the robustness of this finding. 

Last, in another empirical investigation with two studies, metacognitive feelings were again a significant predictor of self-evaluations, and more importantly, they were a significant predictor of evaluative self-efficacy (Puente-Díaz and Cavazos-Arroyo 2022b). Evaluative self-efficacy is a relatively new construct assessing confidence in the ability to evaluate and accurately select the most creative ideas (Steele et al. 2018). Generating ideas with ease informed evaluative self-efficacy, even though it might not be the most reliable or accurate source of information. 

## 7. Summary of Empirical Results

A few tentative conclusions can be drawn from the empirical studies just reviewed. First, given that significant correlations have been found between metacognitive feelings and indicators of metacognitive knowledge and monitoring and control, there is no doubt that feelings play an important role. Metacognitive feelings inform the creative process. Second, creative self-efficacy has a positive relationship with metacognitive feelings. Metacognitive knowledge interacts with metacognitive experiences, facilitating the experience of ease of idea generation when individuals have confidence in their abilities to generate original and effective ideas. Metacognitive feelings inform the process of evaluating and selecting ideas, the relevance of the strengths and weaknesses of the ideas generated, and the belief in the ability to correctly select the most creative ideas. More research is needed to replicate some of these findings and test potential moderators (see Table 1 for a summary of results).

## 8. The Intelligent Use of Feelings to Inform the Creative Process

As stated at the beginning of the article, there has been a heated debate about the wisdom of using feelings when making judgments, decisions (Barrett and Salovey 2002; Vohs et al. 2007), and self-evaluations (Schwarz 2015). In creativity research, scholars have either examined feelings that facilitate or hinder creative performance (e.g., Bass et al. 2008) or have taken a more comprehensive approach and examined how emotion experience and regulation, in the form of emotional intelligence, influence different components of the creative process (e.g., Celume et al. 2020; Ivcevic and Hoffmann 2019). For this conceptual article, the second approach is taken to explore how feelings influence the different components of the creative process. It is important to mention that while the attention given to metacognitive feelings (the focus of this article) has been limited, the attention paid to general feelings and emotions has been more extensive. 

Before beginning to examine how feelings influence the creative process, it is important to explain what better performance or more accurate performance means in terms of idea generation, evaluation, and selection. The issue of intelligent use of feelings and emotions often needs a gold-standard criterion to establish whether feelings help or limit the process of generating, evaluating, and selecting creative ideas. For example, studies have conceptualized better creative potential as the generation of a greater quantity of creative ideas as rated by independent judges. Self-reports of creative performance have also been used, but using judges is considered a more valid and reliable approach. Similarly, studies have examined accurate idea evaluation and selection and conceptualized them as the match between idea generators’ evaluations and selections and judges’ evaluations and selections of the same set of ideas. I only discuss findings consistent with the conceptualization just explained, which includes judges’ evaluations and selections of creative ideas. 

Regarding the intelligent use of feelings, the first possibility is to assess the relationship between feelings and different indicators of idea generation, often referred to as creative potential. Empirical findings on the positive influence of emotional intelligence on creative potential have supported the idea that emotional intelligence is important for generating ideas by influencing attention allocation (Agnoli et al. 2019). In addition, emotion regulation has been shown to positively influence creative performance among career professionals (Parke et al. 2015) and high-school students (Ivcevic and Brackett 2015). It seems that the appropriate use and regulation of feelings have a positive influence on creative potential (Ivcevic and Hoffmann 2019).

A second possibility is that feelings could influence the process of idea evaluation and selection. Regarding the use of feelings when evaluating and selecting ideas, I can use the studies just reviewed to draw some preliminary conclusions, focusing specifically on metacognitive feelings. First, there is no doubt that feelings inform the process of evaluating and selecting ideas. Researchers have found consistent and positive relationships between metacognitive feelings and self-evaluations of the ideas generated (Puente-Díaz and Cavazos-Arroyo 2022a), the relevance of the strengths and weaknesses of the ideas generated (Puente-Díaz and Cavazos-Arroyo 2022b), and confidence in evaluating and correctly selecting the most creative ideas (Puente-Díaz and Cavazos-Arroyo 2022b). 

Regarding the use of feelings in accurate idea evaluation and selection, it is more difficult to reach a conclusion. For example, metacognitive feelings were positively correlated with the overestimation of the creativity of the ideas generated, which then had a negative relationship with accurate idea selection (Puente-Díaz et al. 2021b). Similarly, one study showed that participants evaluated and selected their first idea as the most creative when judges evaluated and selected the third idea generated as the most creative (Puente-Díaz and Cavazos-Arroyo 2022b). In addition, one study showed that participants predicted a decline in the creativity of the idea generated across time, which conflicted with the evaluations made by others showing a positive increase in the creativity of the idea generated (Lucas and Nordgren 2020). Yet, one study showed a positive relationship between metacognitive feelings and accurate idea selection (Puente-Díaz et al. 2021b). Perhaps one way to make sense of these findings is to embrace the concept of creative outcomes as dynamic judgments of potential originality and effectiveness, implying that initial evaluations and selections of ideas might be temporary. If we embrace this view, we must view self-evaluation and selection as a temporary step in the process of idea evaluation and selection. If this is the case, we need to examine, for example, the motivational implications of overestimating the creativity of the ideas generated. Thus far, to my knowledge, this possibility has not been tested empirically.

In sum, more empirical studies are needed to determine with greater certainty when feelings, including metacognitive feelings, limit or facilitate idea generation, evaluation, and selection. From the empirical studies reviewed, we might conclude that metacognitive feelings inform the process of idea generation, selection, and evaluation.

## 9. Future Research Directions

The work on metacognition across different disciplines is abundant. Similarly, the examination of metacognitive feelings is also vast (see Vogl et al. 2021 for a recent conceptual discussion). Yet, in creativity research, most empirical studies have been conducted in the past three or four years. Hence, as is often the case, there are many research areas that have been neglected or received scant attention. We should focus on two areas for future research: (1) the interpretation of metacognitive feelings, and (2) the motivational consequences of metacognitive feelings and idea evaluation and selection. 

The interpretation of metacognitive feelings in the form of ease of idea generation is a relatively neglected area of research. When developing ideas, it is important how individuals feel, and it is also as important how individuals interpret these metacognitive feelings. To simplify things, experiencing ease of idea generation could be interpreted as a sign of high ability or as a sign that the task is easy. This interpretation likely comes from the lay belief that good idea generators should be able to develop ideas with ease. Individuals could also experience difficulty in idea generation. This difficulty could be interpreted as a sign of low ability or as a sign that the task is difficult. Koriat (2006), a meta-memory researcher, was the first to identify and infer how these interpretations influenced study time allocation and judgments of learning. Specifically, he found that sometimes, there was a positive relationship between study time and judgments of learning, and within the same experiment, sometimes the correlation was negative (Koriat 2006). What seems at first paradoxical could be explained by focusing on interpretations of time allocated to studying. Allocating a great deal of time could be interpreted as a sign that participants are ready for the test, and that the information is well-rehearsed and well-learned, leading to high judgments of learning. Individuals might reason that they are going to do well on a test because they allocated a great of time to studying. Conversely, allocating a great deal of time could be interpreted as a sign that participants are not ready for the test and that the information is truly difficult, leading to low judgments of learning. Individuals might reason that because they allocated a great deal of time, they might not do well on a test because the information was really difficult or because of their low ability. Hence, the same experience, allocating time to study, could have different implications for judgments of learning depending on how this presumed difficulty, the allocation of a great deal of time, is interpreted. 

Another example of how the interpretation of metacognitive feelings matters comes from social psychology. One of the first studies on metacognitive feelings found that recalling more instances of assertive behavior led participants to rate themselves as less assertive than recalling fewer instances (Schwarz et al. 1991). This finding is counterintuitive because recalling more instances of assertive behavior should lead to judging one as more assertive due to the greater amount of evidence, but it can be explained by looking at the predominant interpretation of ease or difficulty when generating a few versus many memories of assertive behavior. When asked to recall many instances of assertive behavior, participants reported greater difficulty than participants asked to recall fewer. The dominant interpretation of the reported difficulty could have been that “I am not that assertive since it was hard to generate examples of assertive behavior in my life”. Similarly, participants asked to recall just a few examples felt at ease and interpreted this ease as a sign of overall assertiveness. 

Thus far, the attention paid to metacognitive feelings has been limited, and even more limited has been the attention paid to the interpretation of these feelings. I see great potential for examining the interpretation of metacognitive feelings during the creative process.

As suggested earlier, the concept of potential originality and effectiveness, along with the concept of inconclusiveness of creative outcomes, set the stage for examining additional motivational consequences of metacognitive feelings and idea evaluation and selection. Future research could examine the relationship between metacognitive feelings and persistence, effort, interpretation of negative feedback, and attributions, among others. Similarly, overestimation of the creativity of ideas as informed by metacognitive feelings could lead to higher persistence and effort in idea refinement. Future studies could test this possibility.

In sum, metacognitive feelings inform the creative process of generating, evaluating, and selecting ideas. The use of feelings, as reflected in significant relationships with important outcomes, sometimes leads to adaptive and other times to not-so-adaptive outcomes. Creativity scholars should continue paying attention to metacognition and examining the intelligent use of metacognitive feelings during the creative process. 

## Figures and Tables

**Table 1:** Summary of results.

		Direction of Relationship:		
	Metacognitive feelings correlated with:	Positive or negative	Replication	Articles
1	Self-evaluations of creativity of ideas	Positive	Yes	(Puente-Díaz and Cavazos-Arroyo 2022a)
2	Overestimation of creativity of ideas	Positive	Yes	(Puente-Díaz et al. 2021a)
3	Creative self-efficacy	Positive	Yes	(Puente-Díaz and Cavazos-Arroyo 2020)
4	Relevance of strengths and weaknesses	Positive	Yes	(Puente-Díaz and Cavazos-Arroyo 2022b)
5	Evaluative self-efficacy	Positive	Yes	(Puente-Díaz and Cavazos-Arroyo 2022b)
6	Accurate idea selection	Positive	No	(Puente-Díaz et al. 2021b)

## Data Availability

All datasets are available on request from the first authors of the articles reported in this a review article.

## Note

1 It is worth mentioning that metacognitive feelings have implications for the older and newer definitions of creativity.

## References

[B1-jintelligence-11-00049] Ackerman Rakefet (2019). Heuristic cues for meta-reasoning judgments: Review and methodology. Psychological Topics.

[B2-jintelligence-11-00049] Ackerman Rakefet, Thompson Valery (2017). Meta-Reasoning: Monitoring and control of thinking and reasoning. Trends in Cognitive Sciences.

[B3-jintelligence-11-00049] Agnoli Sergio, Franchin Laura, Rubaltelli Enrico, Corazza Giovanni Emanuele (2019). The emotionally intelligent use of attention and affective arousal under creative frustration and creative success. Personality and Individual Differences.

[B4-jintelligence-11-00049] Ball Linden J., Thompson Valerie A., Ball Linden J., Thompson Valerie A. (2018). Belief bias and reasoning. The Routledge International Handbook of Thinking and Reasoning.

[B5-jintelligence-11-00049] Barrett Lisa F., Salovey Peter (2002). The Wisdom in Feeling: Psychological Processes in Emotional Intelligence.

[B6-jintelligence-11-00049] Baas Matthijs, De Dreu Carsten K. W., Nijstad Bernard A (2008). A meta-analysis of 25 years of mood-creativity research: Hedonic tone, activation, or regulatory focus?. Psychological Bulletin.

[B7-jintelligence-11-00049] Beaty Roger E., Silvia Paul J. (2012). Why do ideas get more creative across time? An executive interpretation of the serial order effect in divergent thinking tasks. Psychology of Aesthetics, Creativity, and the Arts.

[B8-jintelligence-11-00049] Beghetto Ronald A., Madison Ed (2022). Accepting the challenge: Helping schools get smarter about supporting students’ creative collaboration and communication in a changing world. Journal of Intelligence.

[B9-jintelligence-11-00049] Beghetto Ronald A., Karwowski Maciej, Karwowski Maciej, Kaufman James C. (2017). Toward untangling creative self-beliefs. The Creative Self: Effect of Beliefs, Self-Efficacy, Mindset, and Identity.

[B10-jintelligence-11-00049] Celume Macarena-Paz, Goldstein Thalia, Besançon Maud, Zenasni Franck (2020). Developing children’s socio-emotional competencies through drama pedagogy training: An experimental study on theory of mind and collaborative behavior. Europe’s Journal of Psychology.

[B11-jintelligence-11-00049] Corazza Giovanni Emanuele (2016). Potential Originality and Effectiveness: The Dynamic Definition of Creativity. Creativity Research Journal.

[B12-jintelligence-11-00049] De Neys Wim, Franssens Samuel (2009). Belief inhibition during thinking: Not always winning but at least taking part. Cognition.

[B13-jintelligence-11-00049] Dunlosky John, Metcalfe Janet (2009). Metacognition.

[B14-jintelligence-11-00049] Efklides Anastasia (2006). Metacognition and affect: What can metacognitive experiences tell us about the learning process?. Educational Research Review.

[B15-jintelligence-11-00049] Evans Jonathan St B. T. (2020). Hypothetical Thinking: Dual Processes in Reasoning and Judgment.

[B16-jintelligence-11-00049] Flavell John H. (1979). Metacognition and cognitive monitoring: A new area of cognitive-developmental inquiry. American Psychologist.

[B17-jintelligence-11-00049] Ivcevic Zorana, Hoffmann Jennifer D., Kaufman James C., Sternberg Robert J. (2019). Emotions and creativity: From process to person and product. The Cambridge Handbook of Creativity.

[B18-jintelligence-11-00049] Ivcevic Zorana, Brackett Marc (2015). Predicting creativity: Interactive effects of openness to experience and emotion regulation ability. Psychology of Aesthetics, Creativity, and the Arts.

[B19-jintelligence-11-00049] Karwowski Maciej, Lebuda Izabela, Beghetto Ronald A., Kaufman James C., Sternberg Robert J. (2019). Creative Self-Beliefs. The Cambridge Handbook of Creativity.

[B20-jintelligence-11-00049] Karwowski Maciej, Czerwonka Marta, Kaufman James C. (2020). Does intelligence strengthen creative metacognition?. Psychology of Aesthetics, Creativity, and the Arts.

[B21-jintelligence-11-00049] Kaufman James C., Beghetto Ronald A. (2013). In praise of Clark Kent: Creative metacognition and the importance of teaching kids when (not) to be creative. Roeper Review: A Journal on Gifted Education.

[B22-jintelligence-11-00049] Koriat Asher, Uttl Bob, Ohta Nobuo, Siegenthaler Amy L. (2006). Are we frightened because we run away? Some evidence from metacognitive feelings. Memory and Emotion: Interdisciplinary Perspectives.

[B23-jintelligence-11-00049] Koriat Asher (2012). The self-consistency model of subjective confidence. Psychological Review.

[B24-jintelligence-11-00049] Kuhn Deanna (2022). Metacognition matters in many ways. Educational Psychologist.

[B25-jintelligence-11-00049] Lebuda Izabella, Benedek Mathias A systematic framework of creative metacognition.

[B26-jintelligence-11-00049] Lucas Brian J., Nordgren Loran F. (2020). The creative cliff illusion. Proceedings of the National Academy of Sciences of the United States of America.

[B27-jintelligence-11-00049] Metcalfe Janet, Finn Bridgid (2008). Evidence that judgments of learning are causally related to study choice. Psychonomic Bulletin & Review.

[B28-jintelligence-11-00049] Metcalfe Janet, Tulving Endel, Craik Fergus I. M. (2000). Metamemory: Theory and data. The Oxford Handbook of Memory.

[B29-jintelligence-11-00049] Moore Don A., Healy Paul J. (2008). The trouble with overconfidence. Psychological Review.

[B30-jintelligence-11-00049] Moshman David (2015). Epistemic Cognition and Development: The Psychology of Justification and Truth.

[B31-jintelligence-11-00049] Nelson Thomas O., Narens Louis (1990). Metamemory: A theoretical framework and new findings. The Psychology of Learning and Motivation.

[B32-jintelligence-11-00049] Parke Michael R., Seo Myeong-Gu, Sherf Elad N. (2015). Regulating and facilitating: The role of emotional intelligence in maintaining and using positive affect for creativity. Journal of Applied Psychology.

[B33-jintelligence-11-00049] Preiss David D. (2022). Metacognition, mind wandering, and cognitive flexibility: Understanding creativity. Journal of Intelligence.

[B34-jintelligence-11-00049] Puente-Díaz Rogelio, Cavazos-Arroyo Judith (2020). Creative metacognitive feelings as a source of information for creative self-efficacy, creativity potential, intrapersonal idea selection, and task enjoyment. The Journal of Creative Behavior.

[B35-jintelligence-11-00049] Puente-Díaz Rogelio, Cavazos-Arroyo Judith (2022a). Creative self-efficacy and metacognitive feelings as sources of information when generating evaluating, and selecting creative ideas: A metacognitive perspective. The Journal of Creative Behavior.

[B36-jintelligence-11-00049] Puente-Díaz Rogelio, Cavazos-Arroyo Judith (2022b). Evaluative self-efficacy and its potential role in the evaluation and selection of ideas: A metacognitive perspective. Creativity. Theories–Research–Applications.

[B37-jintelligence-11-00049] Puente-Díaz Rogelio, Cavazos-Arroyo Judith, Vargas-Barrera Fernanda (2021a). Metacognitive feelings as a source of information in the evaluation and selection of creative ideas. Thinking Skills and Creativity.

[B38-jintelligence-11-00049] Puente-Díaz Rogelio, Cavazos-Arroyo Judith, Puerta-Sierra Lizbeth (2021b). Idea generation, selection, and evaluation: A metacognitive approach. The Journal of Creative Behavior.

[B39-jintelligence-11-00049] Schwarz Norbert, Bless Herbert, Strack Fritz, Klumpp Gisela, Rittenauer-Schatka Helga, Simons Annette (1991). Ease of retrieval as information: Another look at the availability heuristic. Journal of Personality and Social Psychology.

[B40-jintelligence-11-00049] Schwarz Norbert, Mikulincer Mario, Shaver Phillip R., Borgida Eugene, Bargh John A. (2015). Metacognition. APA Handbook of Personality and Social Psychology: Attitudes and Social Cognition.

[B41-jintelligence-11-00049] Schwarz Norbert, Proust J., Fortier M. (2018). Of fluency, beauty, and truth: Inferences from metacognitive experiences. Metacognitive Diversity. An Interdisciplinary Approach.

[B42-jintelligence-11-00049] Sidi Yael, Torgovitsky Ilan, Soibelman Daniela, Miron-Spektor Ella, Ackerman Rakefet (2020). You may be more original than you think: Predictable biases in self-assessment of originality. Acta Psychologica.

[B43-jintelligence-11-00049] Stanovich Keith (2011). Rationality And the Reflective Mind.

[B44-jintelligence-11-00049] Steele Logan M., Johnson Genevieve, Medeiros Kelsey E. (2018). Looking beyond the generation of creative ideas: Confidence in evaluating ideas predicts creative outcomes. Personality and Individual Differences.

[B45-jintelligence-11-00049] Stein Morris I. (1953). Creativity and Culture. The Journal of Psychology.

[B46-jintelligence-11-00049] Tarricone Pina (2011). The Taxonomy of Metacognition.

[B47-jintelligence-11-00049] Tversky Amos, Kahneman Daniel (1973). Availability: A heuristic for judging frequency and probability. Cognitive Psychology.

[B48-jintelligence-11-00049] Unkelbach Christian, Greifeneder Rainer, Unkelbach Christian, Greifender Rainer (2013). A general model of fluency effects in judgment and decision making. The Experience of Thinking: How the Fluency of Mental Processes Influences Cognition and Behaviour.

[B49-jintelligence-11-00049] Vogl Elisabeth, Reinhard Pekrun, Kristina Loderer, Moraitou Despina, Metallidou Panayiota (2021). Epistemic emotions and metacognitive feelings. Trends and Prospects in Metacognition Research across the Life Span: A Tribute to Anastasia Efklides.

[B50-jintelligence-11-00049] Vohs Kathleen D., Baumeister Roy F., Loewenstein George (2007). Do Emotions Help or Hurt Decision-Making?: A Hedgefoxian Perspective.

